# High Serum Uric Acid and High-Sensitivity C Reactive Protein Concentrations Predict Three-Year Cardiovascular Mortality in Patients Treated With Continuous Ambulatory Peritoneal Dialysis

**DOI:** 10.7759/cureus.17900

**Published:** 2021-09-12

**Authors:** Quyen Dao Bui Quy, Tuan Pham Ngoc Huy, Tuan Nguyen Minh, Loc Nguyen Duc, Tuan Nguyen Minh, Kien Nguyen Trung, Tien Tran Viet, Quyet Do, Thang Le Viet

**Affiliations:** 1 Nephrology, Cho Ray Hospital, Ho Chi Minh, VNM; 2 Intensive Care Unit, Trung Vuong Hospital, Ho Chi Minh, VNM; 3 Hemodialysis, Cho Ray Hospital, Ho Chi Minh, VNM; 4 Nephrology, An Sinh Hospital, Ho Chi Minh, VNM; 5 Urology, E Hospital, Hanoi, VNM; 6 Hematology and Blood Transfusion, Military Hospital 103, Hanoi, VNM; 7 Director, Military Hospital 103, Hanoi, VNM; 8 Director, Vietnam Military Medical University, Hanoi, VNM; 9 Nephrology and Hemodialysis, Military Hospital 103, Hanoi, VNM

**Keywords:** capd, uric acid, hs-crp, cardiovascular-related mortality, predicting

## Abstract

Aims: This study aims to access the predicting value of serum uric acid (UA) and high-sensitivity C reactive protein (hs-CRP) concentration on three-year cardiovascular-related mortality in patients performing continuous ambulatory peritoneal dialysis (CAPD).

Methods: A total of267 CAPD patients [150 male (56.2%); mean age 48.93 ± 13.58 years] were included in our study. All patients had measured serum UA and hs-CRP concentration. A high-sensitivity particle-enhanced immunoturbidimetric assay determined serum hs-CRP; serum UA levels were determined using an enzymatic colorimetric assay. All patients were followed for three years to detect cardiovascular-related mortality by cardiologists and stroke specialists.

Results: Mean serum UA level was 415.16 ± 84.28 µmol/L, 58.4% of patients had increased serum UA level. Median serum hs-CRP level was 2 (1-4) mg/L, 12.4% of patients had increased serum hs-CRP level. During 36 months of follow-up, 41 patients (15.4%) had cardiovascular-related mortality. The results of Cox proportional hazards regression showed that hypertension, diabetes, high serum UA and hs-CRP were risk factors that related to cardiovascular-related mortality (p<0.05). The* *receiver operating characteristic (ROC) curve and Kaplan-Meier analysis results showed that UA and hs-CRP level had predictive value for three-year cardiovascular-related mortality in CAPD patients [uric acid: area under the curve (AUC)=0.822; hs-CRP: AUC=0.834, p < 0.001].

Conclusion: High serum UA and hs-CRP levels were predictive factors of cardiovascular-related mortality in CAPD patients.

## Introduction

Peritoneal dialysis (PD) is one of the most common kidney replacement therapies. Peritoneal dialysis is performed by using a catheter to infuse sterile solution into the peritoneal cavity. The peritoneum is used as an exchange filter to remove solutes [1-3]. PD consists of two methods: continuous ambulatory and automated PD, in which continuous ambulatory PD (CAPD) is widely used in Vietnam. A mortality rate in CAPD from 8.3% to 39.2% was reported in previous studies [4-6]. Prolonged use of CAPD is related to a higher mortality rate [4,6], in which mortality due to cardiovascular events was behind infection-related ones.

C-reactive protein (CRP) is synthesized mainly in the liver and released into the bloodstream in response to an acute phase of tissue damage or infection. Many studies have also shown that some factors such as for overweight, elderly, hypertension, diabetes, smoking, and other cardiovascular risks also affect hs-CRP levels [7-10]. In PD patients, elevated hs-CRP levels are related to higher cardiovascular events and mortality [11-13]. Uric acid (UA) is a purine metabolism production in humans. Elevated serum uric acid concentration is related to the appearance of cardiovascular events such as hypertension, coronary heart disease, stroke and the elevation of mortality rate [14-16]. High serum UA was also confirmed as one of the risk factors for cardiovascular events and PD patients' mortality [17-20]. PD is a typical renal replacement therapy in Vietnam. Up to now, there is no research in Vietnam on prevalence as well as risk factors relating to cardiovascular-related mortality in CAPD patients. For these reasons, we performed this study to access the predictive values of serum UA and hs-CRP for cardiovascular-related mortality during three years follow-up in Vietnamese CAPD patients.

The Ethical Committee of Cho Ray Hospital has approved this study (No.1378/QĐ/BVCR). All human procedures followed the local ethical standards and the Helsinki Declaration of 1975 (revised in 2008). All patients signed written informed consent before participating in this study.

## Materials and methods

Study design

**Figure 1:**
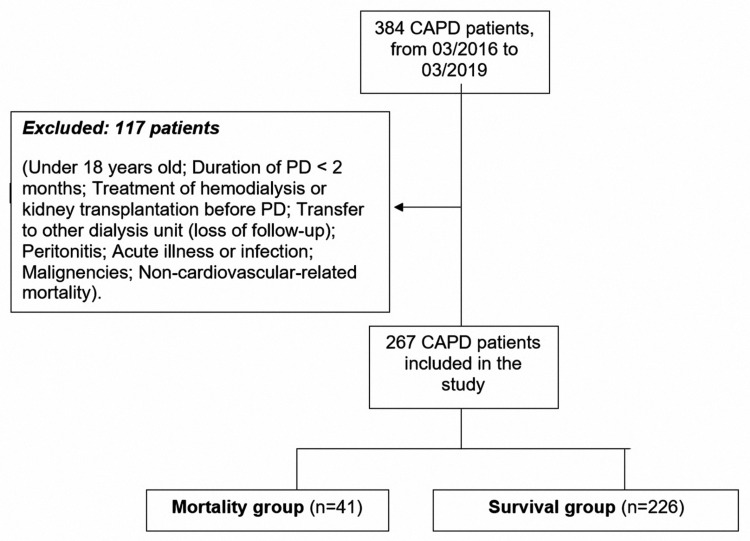
Study population and analyzed groups PD: peritoneal dialysis, CAPD: continuous ambulatory peritoneal dialysis

A total of 384 CAPD patients participated in our study at the Department of Nephrology, Cho Ray Hospital, Ho Chi Minh City, Vietnam, from March 2016 to March 2019. We excluded 117 patients who had one of the exclusion criteria such as age under 18 years old, duration of PD < two months, on hemodialysis treatment or who had kidney transplantation before initiation of PD, peritonitis, acute illness, a sign of infection (hs-CRP ≥ 10 mg/L; white blood cell count ≥ 10 G/L; or neutrophil percentage ≥ 70%), malignancies, transfer to other dialysis units (loss of follow-up), non-cardiovascular-related mortality, etc. The remaining 267 patients (150 male and 117 female) were selected and signed informed consent before participation in this study. A flowchart of the study is presented in Figure 1.

All patients had 24-hour urine volume collected and measured. Creatinine clearance (CCr) week, Kt/V week, and peritoneal equilibration test (PET) were also measured. We used the dialysate to the plasma concentration ratio of creatinine (D/Pcreat) to evaluate the peritoneal small solute transport rate. Based on Twardowski's 1987 classification, PET is classified into four levels [21] as follows: high (H: D/Pcreat ≥ 0.81), high-average (HA: D/Pcreat 0.65-0.80), low-average (LA: D/Pcreat 0.51-0.64), and low (L: D/Pcreat ≤0.5) transporters. 

We collected all demographic data in baseline time (includes: age, gender, time of peritoneal dialysis, measuring height, weight, calculating BMI, etc.). Blood was taken to measure necessary hematological and biochemical indices by standard laboratory methods. Serum samples were collected by standard venepuncture during the first annual visit post-HTx, frozen samples (stored at −80°C) were available for 267 patients. All biochemical analyses, including glucose, creatinine, urea, albumin, protein, and electrolyte parameters, were measured by an automatic analyzer. Levels of serum hs-C-reactive protein were determined by a high-sensitivity particle-enhanced immunoturbidimetric assay [Tina-quant C-reactive protein (Latex) High Sensitive (Roche Diagnostics, Basel, Switzerland)] with an inter-assay variance <10% in the range 0.1-300 mg/L. Serum uric acid levels were determined using an enzymatic colorimetric assay (Roche Uric Acid Plus; Roche Diagnostics). The measuring range was 12-3720 µmol/L, with imprecision coefficients of variation of < 2.3%. 

Diabetes was diagnosed according to the patient's history of diabetes, or two subsequent analyzes showed fasting blood glucose levels ≥ 7.0 mmol/L. Hypertension was diagnosed in patients who regularly used antihypertensive drugs to control blood pressure or had at least two times blood pressure ≥140/90 mm Hg. Anemia was diagnosed when hemoglobin < 130g/L in males or < 120 g/L in females. We calculated residual renal creatinine clearance (ClCr) as the average of renal urea and creatinine clearances, indexing to body surface area [22]. Lipid disorder was diagnosed based on the 2013 KDIGO guideline [23].

Follow-up and outcomes

We followed up to detect death from cardiovascular causes (Cardiovascular cause mortality in our patients was defined as death attributable to myocardial ischemia and infarction, heart failure, cardiac arrest, or cerebrovascular accident [24]) for 36 months. All of these events were confirmed by cardiologists and stroke specialists.

Statistical analyses

We processed and analyzed data by the Statistical Package for Social Science (SPSS) version 20.0 (IBM Corp., Armonk, NY, USA). We used Student's t-test to compare two mean values of continuous normal-distribution data and Mann-Whitney U test to compare two median values of non-normal distribution data. We used the Chi-square test to compare two groups of categorical data. The Cox proportional hazards regression analysis was performed to determine risk factors of mortality after three years' follow-up. We performed the receiver operating characteristic (ROC) curves model and the Kaplan-Meier analysis to determine the mortality predicting the value of hs-CRP, uric acid, and prealbumin after three years' follow-up. A p-value <0.05 was considered significant.

## Results

In Table 1, the patient's mean age was 48.93 ± 13.58 years (56.2% male, 16.1% diabetic mellitus (DM), and 83.1% hypertension). The median PD duration was 21 months. The proportion of lost residual kidney function and anemia was 71.9% and 95.5%, respectively. The mean serum UA was 415.16 ± 84.28 µmol/L, with a 58.4% increase. The median serum hs-CRP was 2.0 mg/L, with a 12.4% increase, and 15.4% of patients died cardiovascular cause during three years.

**Table 1:** Baseline demographic and laboratory characteristics of patients PD: Peritoneal Dialysis; BMI: Body Mass Index; PET: Peritoneal Equilibration Test; H: High; HA: High-Average; LA: Low-Average; L: Low; CCr: Creatinine clearance; WBC: White Blood Cell; hs-CRP: high sensitive C Reactive Protein.

Clinical characteristics and laboratory parameters	Mean ± SD/ Median	n,%
Ages (years)	48.93 ± 13.58	N/A
Number of males (n,%)	N/A	150 (56.2)
PD duration (month)	21 (10 – 41)	N/A
Hypertension (n,%)	N/A	222 (83.1)
Diabetic mellitus (n,%)	N/A	43 (16.1)
BMI (kg/m^2^)	21.21 ± 2.95	N/A
BMI < 18.5 (n,%)	N/A	44 (16.5)
BMI: 18.5 – 22.9 (n,%)	N/A	160 (59.9)
BMI ≥ 23 (n,%)	N/A	63 (23.6)
Residual kidney function (n,%)	N/A	75 (28.1)
24-hours urine volume (ml)	180 (130 – 500)	N/A
D4/P	0.7 ± 0.08	N/A
PET H (n,%)	N/A	20 (7.5)
PET HA (n,%)	N/A	166 (62.2)
PET LA (n,%)	N/A	78 (29.2)
PET L (n,%)	N/A	3 (1.1)
Serum urea (mmol/L)	19.34 ± 6.14	N/A
Creatinine (µmol/L)	774.37 (654.9 – 955.8)	N/A
Kt/V	1.98 ± 0.3	N/A
Total CCr (L/week/1.73m^2^)	62.6 ± 9.32	N/A
Hemoglobin (g/L)	100.14 ± 17.01	N/A
Anemia (n,%)	N/A	255 (95.5)
WBC (G/L)	6.88 ± 1.49	N/A
Neutrophil (G/L)	61.36 ± 8.52	N/A
Glucose (mmol/L)	4.22 (3.77 – 4.83)	N/A
Na+ (mmol/L)	136.92 ± 3.75	N/A
K+ (mmol/L)	3.68 ± 0.78	N/A
Ca++ (mmol/L)	2.05 ± 0.3	N/A
Protein (g/dL)	6.51 ± 0.7	N/A
Albumin (g/dL)	3.68 ± 0.48	N/A
Prealbumin (mg/dL)	0.34 ± 0.08	N/A
Uric acid (µmol/L)	415.16 ± 84.28	N/A
Increase uric acid (n,%)	N/A	156 (58.4)
hs-CRP (mg/L)	2 (1 – 4)	N/A
Increase hs-CRP (n,%)	N/A	33 (12.4)
Mortality (n,%)	N/A	41 (15.4)

In mortality patients, the ratio of diabetes, the mean UA, and the median hs-CRP concentration was significantly higher than those of the survival group, while the mean prealbumin concentration was lower than those of the survival group (p< 0.05) (Table 2).

**Table 2:** Comparison of demographic and laboratory characteristics between mortality and survival group PD: Peritoneal Dialysis; BMI: Body Mass Index; PET: Peritoneal Equilibration Test; H: High; HA: High-Average; LA: Low-Average; L: Low; CCr: Creatinine clearance; WBC: White Blood Cell; hs-CRP: high sensitive C Reactive Protein.

Clinical characteristics and laboratory parameters	Mortality group (n=41)	Survival group (n=226)	p
Ages (years)	47.98 ± 12.91	49.1 ± 13.72	0.628
Number of male (n,%)	27 (65.9)	123 (54.4)	0.175
PD duration (month)	24 (15.5 – 43.5)	19 (9 – 39.25)	0.22
Hypertension (n,%)	30 (73.2)	192 (85)	0.064
Diabetic mellitus (n,%)	17 (41.5)	26 (11.5)	< 0.001
BMI (kg/m^2^)	22.33 ± 3.78	21 ± 2.74	0.038
BMI < 18.5	5 (12.2)	39 (17.3)	0.04
BMI: 18.5 – 22.9	20 (48.8)	140 (61.9)	
BMI ≥ 23	16 (39)	47 (20.8)	
24-hour urine volume (ml)	165 (117.5 – 600)	180 (130 – 500)	0.681
Residual kidney function	12 (29.3)	63 (27.9)	0.855
D4/P	0.71 (0.62 – 0.75)	0.73 (0.62 – 0.77)	0.187
PET H (n,%)	3 (7.3)	17 (7.5)	0.529
PET HA (n,%)	22 (53.7)	144 (63.7)	
PET LA (n,%)	15 (36.6)	63 (27.9)	
PET L (n,%)	1 (2.4)	2 (0.9)	
Serum urea (mmol/L)	21.62 ± 5.13	18.93 ± 6.23	0.01
Creatinine (µmol/L)	831.9 (699.15 – 982.35)	761.1 (643.61 – 955.8)	0.226
Kt/V	1.95 ± 0.33	1.99 ± 0.3	0.47
Total CCr (L/week/1.73m^2^)	61.45 ± 11.29	62.81 ± 8.93	0.393
Hemoglobin (g/L)	104.16 ± 12.5	99.41 ± 17.63	0.1
Anemia (n,%)	40 (97.6)	215 (95.1)	0.49
WBC (G/L)	7.03 ± 1.56	6.85 ± 1.48	0.472
Neutrophil (%)	61.1 ± 10.4	61.4 ± 8.16	0.836
Glucose (mmol/L)	4.22 (3.75 – 4.75)	4.19 (3.81 – 4.84)	0.898
Na+ (mmol/L)	137.09 ± 3.44	136.89 ± 3.8	0.745
K+ (mmol/L)	3.87 ± 0.78	3.64 ± 0.78	0.094
Ca++ (mmol/L)	2.09 ± 0.36	2.04 ± 0.29	0.372
Protein (g/dL)	6.59 ± 0.88	6.5 ± 0.66	0.448
Albumin (g/dL)	3.77 ± 0.45	3.67 ± 0.49	0.222
Prealbumin (mg/dL)	0.3 ± 0.07	0.34 ± 0.08	0.005
Uric acid (µmol/L)	502.7 ± 105.63	399.28 ± 69.02	< 0.001
Increase uric acid (n,%)	35 (85.4)	121 (53.5)	< 0.001
hs-CRP (mg/L)	5.1 (3.1 – 5.65)	2 (1 – 3.6)	< 0.001
Increase hs-CRP (n,%)	21 (51.2)	12 (5.3)	< 0.001

The results of Cox proportional hazards regression in Table 3 showed that various risk factors were related to mortality, including hypertension, diabetes, high serum UA and hs-CRP (p<0.05).

**Table 3:** Cox proportional hazards regression analysis to determine risk factors of mortality hs-CRP: high sensitive C Reactive Protein.

Variable	HR	95% Cl	p
Hypertension	2.051	1.011 – 4.157	0.046
Diabetes	0.361	0.187 – 0.698	0.002
Increase uric acid (µmol/L)	0.282	0.117 – 0.679	0.005
Increase hs-CRP (mg/L)	0.091	0.047 – 0.175	< 0.001

**Figure 2:**
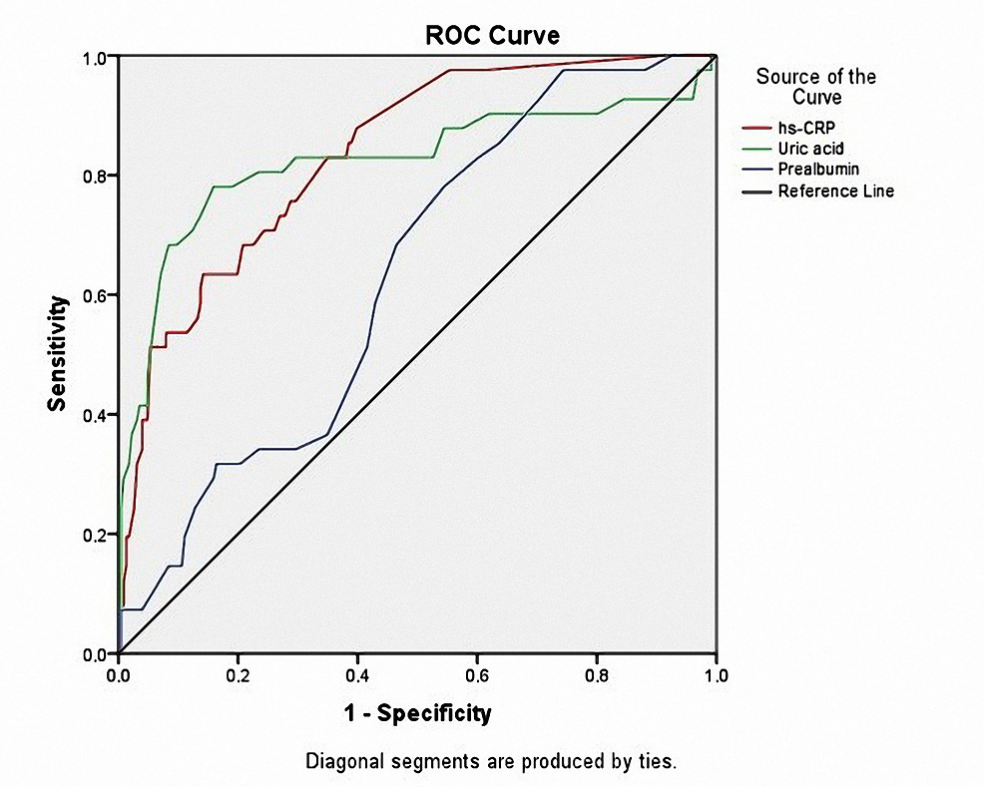
Receiver operating characteristics (ROC) curve of prealbumin, hemoglobin, serum UA, and hs-CRP to predict mortality. UA: uric acid; hs-CRP: high-sensitivity C reactive protein

Based on ROC curve analysis results in Figure 2, some factors predicted cardiovascular-cause mortality, in which serum UA level and hs-CRP had a substantial value, p<0.001 (hs-CRP: AUC=0.834, p<0.001, cut-off value = 4.05 (mg/L), Se=63.4%, Sp=85.8%; Uric acid: AUC=0.822, p<0.001, cut-off value = 467.07 (µmol/L), Se=78%, Sp= 84.1%; Prealbumin: AUC=0.631, p=0.008, cut-off value = 0.365 (mg/dL), Se=78%, Sp=45.6%).

**Figure 3:**
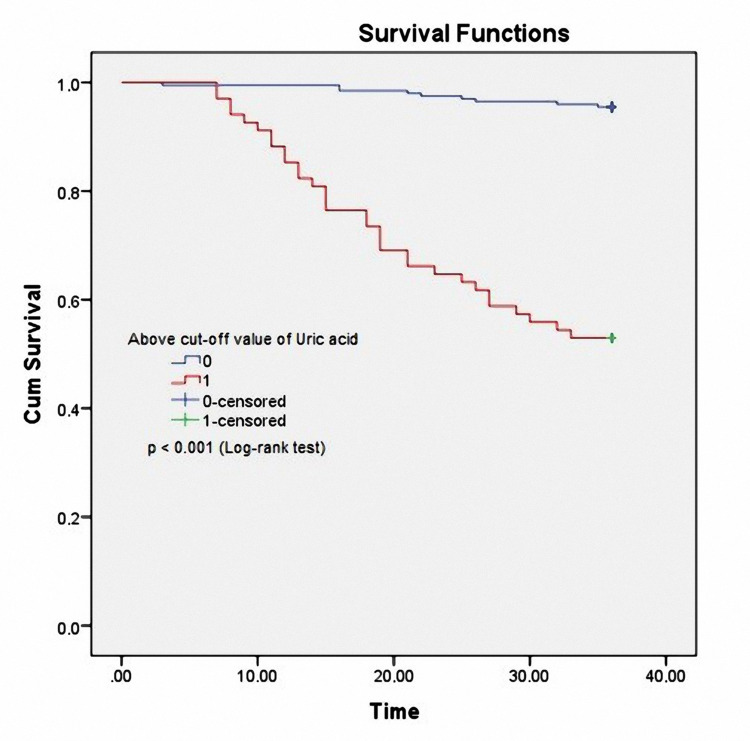
Kaplan–Meier analysis for all CAPD patients' mortality, classified according to UA level UA: uric acid; CAPD: continuous ambulatory peritoneal dialysis

The Kaplan-Meier analysis in Figure 3 showed that patients with higher serum UA level (UA ≥ 467.07 µmol/L: red line) had a significantly higher mortality rate than those with lower UA level (UA < 467.07 µmol/L: violet line) (Log-rank test, p<0.001).

**Figure 4:**
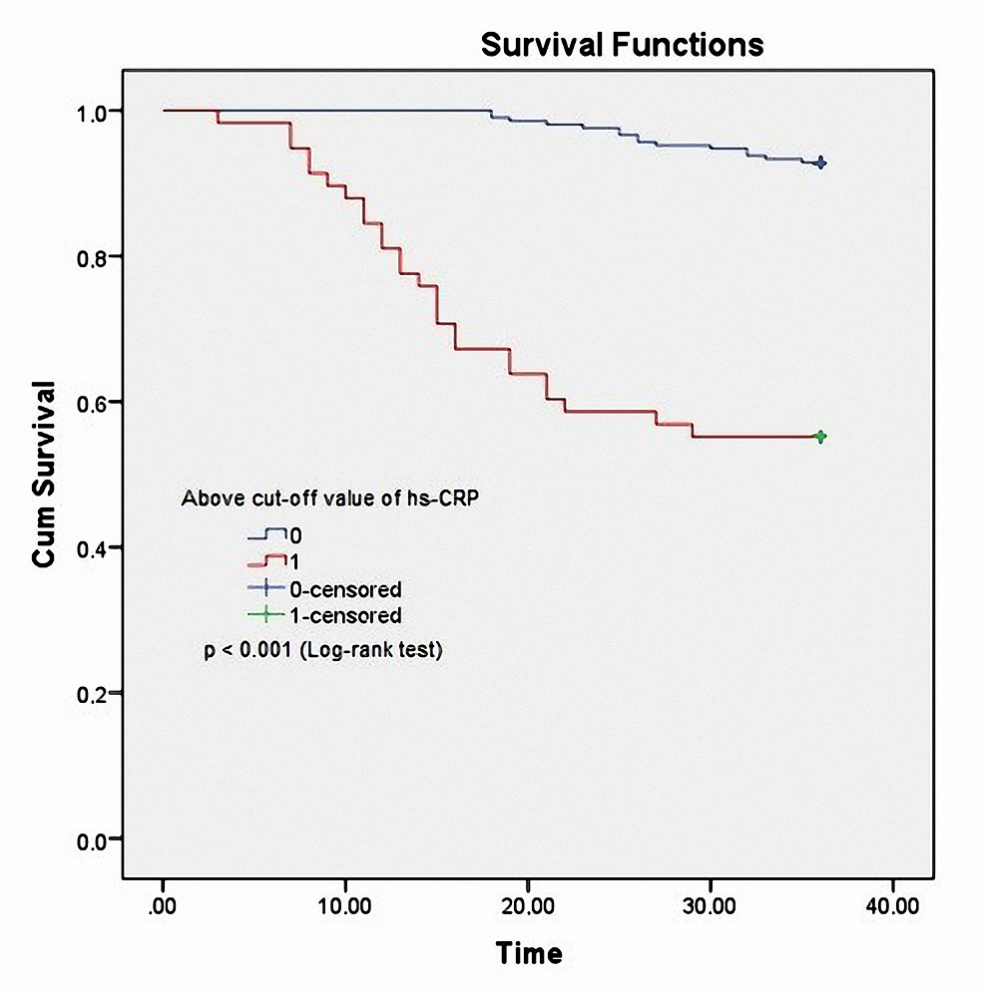
Kaplan–Meier analysis for all CAPD patients' mortality, classified according to serum hs-CRP concentration CAPD: continuous ambulatory peritoneal dialysis; hs-CRP: high-sensitivity C reactive protein

Similar to UA, the Kaplan-Meier analysis showed that patients with higher serum hs-CRP level (hs-CRP ≥ 4.05 µmol/L: red line) had a significantly higher mortality rate than those with lower hs-CRP level (hs-CRP < 4.05 µmol/L: violet line) (Log-rank test, p<0.001) (Figure 4).

## Discussion

The concentration of serum UA, hs-CRP, and ratio of mortality

An increase in serum UA was reported in previous studies [17-20,25]. In non-dialysis chronic kidney disease (CKD) patients, the hyperuricemia rate ranged from 40 to 70% [26-28]. In dialysis patients, the ratio of hyperuricemia was 34.4% [25] and is related to the longer duration of hemodialysis or peritoneal dialysis [27]. Peritoneal membrane plays an essential role in removing UA from circulation in PD patients [29,30]. UA is a small molecular solute in its ionized form and is highly hydrophilic. UA is rapidly diffused through the peritoneal membrane to be efficiently purified by PD [31]. It explains that elevated serum UA ratio in dialysis patients was lower than non-dialysis CKD ones. As for serum UA, an increase of serum hs-CRP was reported in PD patients [7-10]. Fine [32] found 31% of PD patients with increasing serum CRP. The majority of the unexplained increase in CRP levels in peritoneal dialysis patients is associated with atherosclerosis [32]. Atherosclerosis is a chronic inflammatory disease [33]. Atherosclerosis begins with a fatty streak, an accumulation of lipid-laden foam cells in the intimal layer of the artery. Lipid retention is the first step in atherosclerosis's pathogenesis, which is followed by chronic inflammation at susceptible sites in the artery walls [33,34].

The ratio of cardiovascular-cause mortality was 15.4% in our study (Table 1) during three years of follow-up. Ye et al. [4] announced prevalence of all-cause mortality was 19.8%, cardiovascular-related death was 11.13% in 1321 PD patients with a mean age of 48.1 ± 15.3 years, and 58.7% of males. The proportion of diabetes and history of cardiovascular diseases (CVD) was 23.5% and 36.3%, respectively. Causes of death are almost related to peritonitis, CVD, malignant, cachexia in PD patients. Tsujikawa et al. [35] reported that the all-cause mortality ratio was 22.8%, in which cardiovascular-related death reached 7.9% (48/606 patients). Dong et al. [18] showed that the ratio of cardiovascular-related mortality was 10.2% (231 patients) after evaluation in 2264 PD patients (mean age of 58.1 ± 15.5 years). Thus, mortality from cardiovascular causes is common and accounts for a significant PD patient rate, just behind the infection's death rate.

Relationship between UA, hs-CRP, and cardiovascular-related mortality

Our results showed some characteristics of PD patients relating to mortality, including hypertension, overweight, and obesity, DM, hypoprealbuminia, and anemia (Table 2, 3). Some previous studies have also confirmed the fact [36-39]. Thus, hypertension, DM, overweight, and obesity are risk factors and causes of many complications such as cerebrovascular disorders, myocardial ischemia, and peripheral artery disease. Elevated serum hs-CRP levels are a consequence of atherosclerosis, which is triggered by local inflammation in the arterial wall. On the other hand, serum hs-CRP increased closely to malnutrition, which occurred in 30-50% of PD patients [40,41]. Malnutrition directly leads to infection and death through weight loss, impaired immunity, and mucosal damage, which increases the risk of pathogen infiltration and complicates in patients treated with PD [41].

In the study, we found a relationship between high serum UA; hs-CRP and cardiovascular-related mortality. Our results also showed that UA and hs-CRP had good values for predicting cardiovascular-related mortality after 36 months of follow-up (Figure 2, 3, 4). UA is a potent antioxidant. Elevated UA levels can decrease nitric oxide (NO), endothelial disorders, atherosclerosis, etc. [42,43]. UA is an inexpensive, easy-to-perform biomarker that makes it very popular in clinical practice to assess cardiovascular risk factors. hs-CRP is a biological marker for evaluating nonspecific inflammation and has been widely used as a cardiovascular risk factor [44]. hs-CRP itself mediates atherothrombosis. Many recent studies have shown that hs-CRP is directly related to and predicts cardiovascular events [16,44,45]. UA and hs-CRP have been confirmed to predict all-cause mortality as well as infection-related mortality in PD patients in some previous studies [4,5,14,15]. Our results also demonstrate that clinicians can use serum UA and hs-CRP to predict cardiovascular-related mortality in CAPD patients.

Limitations

We think that the study results have met our research hypothesis, which is to use serum UA and hs-CRP (indices easily collected in clinical practice) to predict cardiovascular-related mortality in CAPD patients. However, there are still some limitations in this study. Firstly, this is a single-center descriptive observational study, so the characteristics of Vietnamese CAPD patients have not been fully described. Nextly, we did not analyze the relationship between residual renal function, peritoneal function, peritoneal filtration efficiency, and peritonitis with cardiovascular-related mortality.

## Conclusions

In conclusion, the ratio of cardiovascular-related mortality was 15.4%. The mean UA and the median hs-CRP concentration were significantly higher in the mortality group than in the survival group. Hypertension, diabetes, high serum UA and hs-CRP were independent risk factors related to mortality in CAPD patients.

High serum UA and hs-CRP concentrations were good predictors of cardiovascular-related mortality in CAPD patients based on the ROC curve model and Kaplan-Meier analysis.
